# The Psychological Experience of Frontline Perioperative Health Care Staff in Responding to COVID-19: Qualitative Study

**DOI:** 10.2196/27166

**Published:** 2021-09-29

**Authors:** Toni Withiel, Elizabeth Barson, Irene Ng, Reny Segal, Daryl Lindsay Goulding Williams, Roni Benjamin Krieser, Keat Lee, Paul Mario Mezzavia, Teresa Sindoni, Yinwei Chen, Caroline Anne Fisher

**Affiliations:** 1 Department of Allied Health Royal Melbourne Hospital Parkville Australia; 2 Department of Anaesthesia and Pain Management Royal Melbourne Hospital Parkville Australia; 3 Department of Medicine University of Melbourne Parkville Australia; 4 The Melbourne Clinic Richmond Australia

**Keywords:** COVID-19, perioperative, mental health, qualitative, grief, psychology, health care worker, experience, hospital, trauma, thematic analysis, interview

## Abstract

**Background:**

The rapid spread of the novel coronavirus (COVID-19) has presented immeasurable challenges to health care workers who remain at the frontline of the pandemic. A rapidly evolving body of literature has quantitatively demonstrated significant psychological impacts of the pandemic on health care workers. However, little is known about the lived experience of the pandemic for frontline medical staff.

**Objective:**

This study aimed to explore the qualitative experience of perioperative staff from a large trauma hospital in Melbourne, Australia.

**Methods:**

Inductive thematic analysis using a critical realist approach was used to analyze data from 9 semistructured interviews.

**Results:**

Four key themes were identified. *Hospital preparedness* related to the perceived readiness of the hospital to respond to the pandemic and encompassed key subthemes around communication of policy changes, team leadership, and resource availability. Perceptions of readiness contributed to the perceived *psychological impacts of the pandemic*, which were highly varied and ranged from anger to anxiety. A number of *coping strategies* were identified in response to psychological impacts which incorporated both internal and external coping mechanisms. Finally, *adaptation with time* reflected change and growth over time, and encompassed all other themes.

**Conclusions:**

While frontline staff and hospitals have rapidly marshalled a response to managing the virus, relatively less consideration was seen regarding staff mental health in our study. Findings highlight the vulnerability of health care workers in response to the pandemic and reinforce the need for a coordinated approach to managing mental health.

## Introduction

The SARS-CoV-2 (herein referred to as COVID-19) pandemic has profoundly changed the fabric of society and presents unparalleled psychosocial and economic challenges at a global scale. Originally identified in the Chinese city of Wuhan in December 2019 [1], the novel pneumonia has spread rapidly around the world. As of December 3, 2020, the World Health Organization [2] reported 64.2 million confirmed cases of COVID-19 with 1.49 million mortalities. On March 11, 2020, the WHO declared COVID-19 as a pandemic with subsequent lockdown measures implemented in Australia. The state of Victoria in Australia has been significantly impacted by COVID-19 with 74.4% of all cases originating in Victoria as of September 14, 2020. As a consequence of the “second wave” of cases, Victorian hospitals were forced to rapidly enact their emergency protocols.

At the Royal Melbourne Hospital, decision making in response to the pandemic occurred in an expedited fashion, often with limited information available. As a consequence of the pandemic, the hospital shifted to a COVID surge planning strategy, developed from late February 2020. Staff communication avenues for the strategy included a hospital Workplace (corporate social media), dedicated COVID-19 information page (established on February 27, 2020), daily dissemination of information in the heads of department hospital huddle, hospital mangers’ briefings (commencing March 23, 2020), and whole-of-hospital Workplace Chat sessions (commencing April 2, 2020). Personal protective equipment (PPE) requirement guidelines were developed and varied according to information available from the Department of Health and Human Services. These changes were disseminated to staff, as available.

At the frontline of the pandemic are health care workers (HCWs) who play a direct role in the diagnosis, treatment, and care of patients diagnosed with COVID-19. The virulence and mortality rate of the virus, along with depletion of PPE and rapidly evolving workplace roles, place HCWs at a distinct risk of experiencing psychological distress [3]. These risks are further exacerbated by the strict lockdown measures imposed in Victoria, with the psychosocial implications of prolonged isolation remaining largely unknown [4]. Existing literature highlights the psychological vulnerability of HCWs who knowingly jeopardize their own health to uphold the health and well-being of others [5]. Indeed, 3573 Victorian HCWs became infected and a significant proportion of these cases were within the hospital setting [6]. Unsurprisingly, an increasing number of quantitative studies have described elevated incidence of depression, anxiety, insomnia, and distress among HCWs exposed to COVID-19 through their work [3,7-9]. Pooled incidence rates from a recent meta-analysis found elevated levels of anxiety, depression, and insomnia, at levels of 23.2%, 22.8%, and 38.9%, respectively, among HCWs exposed to COVID-19 [7]. Simply being a health care worker has been identified as an independent predictor of psychological distress [3,10]. Additional risk factors have been reported to include being female and being a nurse [7]. Further, contributing factors include speculation around modes of transmission, how quickly the virus has spread, and a lack of definitive treatment protocols or vaccinations [11]. Taken together, it is clear that COVID-19 is having profound mental health impacts on HCWs across a broad range of settings.

To date, the vast majority of studies conducted into the mental health impacts of COVID-19 on HCWs has been quantitative in nature. While affording well-documented strengths in internal validity and replicability, the use of quantitative approaches alone may be insufficient to explore the complex nature of psychological distress during this unprecedented time. A recent position paper summarizing the priorities identified by 24 world-leading experts further reinforced the importance of lived experience in characterizing mental health implications [4]. Hence, qualitative approaches represent an important avenue to characterizing the richness and diversity of HCW experiences.

The limited number of qualitative studies that have been conducted to date have revealed a number of themes. Sun and colleagues [12] examined the psychological experiences of 20 nurses caring for patients with COVID-19 in China. Four themes were generated in that study. The theme “significant amounts of negative emotions at an early stage” highlighted the high degree of exhaustion, helplessness, and unfamiliarity in navigating through the early stages of the pandemic. *Self-coping styles* was also constructed as a theme with participants describing the adoption of a range of coping mechanisms to psychologically adapt to the pandemic, ranging from avoidance to relaxation and humor. Growth under stress was identified among participant accounts, with many identifying a new found appreciation and gratitude for family, social supports, and health. The final theme reflected the juxtaposition of having both positive and negative emotions occur simultaneously. Similar themes were identified in a qualitative study of 30 frontline health care nurses in Wuhan, China [13], where both positive and negative psychological consequences were described.

Using thematic analysis, Munawar and Choudhry [14] explored challenges and coping mechanisms among HCWs in Pakistan. Participants reported limiting media exposure and limiting disclosing of work responsibilities to close others as key coping tools. However, a number of culture-specific coping methods were noted, including religious coping and faith-based practices. Unique challenges around denial by religious scholars were also described, with participants reporting frustration at public noncompliance. Taken together, findings highlight the religious and culture-specific experiences faced by HCWs during the COVID-19 pandemic.

To date, no studies have examined the experience of frontline HCWs in psychologically responding to, and dealing with, the COVID-19 pandemic in an Australian context. This study aims to qualitatively explore the psychological impact of the COVID-19 pandemic among frontline HCWs in the perioperative department of a large, tertiary trauma hospital in Melbourne, Australia. Perioperative teams include HCWs that care for patients before, during, and after a surgery or interventional procedures. Included in this HCW cohort are nurses, theater managers, surgeons, anesthetists, surgical and esthetic trainees, theater technicians, and radiographers. In this area of health care, there is a high throughput of patient turnover, frequent aerosolizing procedures, and close contact with patients, some of them with unknown COVID-19 status and with aerosolizing-generating behaviors.

## Methods

### Participants and Setting

Reported in this paper are the data from the qualitative component of a mixed-methods research study evaluating the psychological impact of the COVID-19 pandemic on perioperative staff. A longitudinal quantitative data collection is ongoing via a 4-weekly survey, and the results of this will be reported elsewhere, after study completion. Participants were frontline HCWs employed in the perioperative department of the Royal Melbourne Hospital. All perioperative staff were eligible to participate. Participants were invited by email to register their interest for the study online via a link on their email at the time points of months 4, 5, and 6 of the 7-month-long quantitative study. All participants who registered “interested” were contacted via their preferred method (email or telephone) and provided with the participant information and consent form about the study. Consenting staff were then scheduled for interviews.

Recruitment for the qualitative arm of this study took place in late August 2020, coinciding with the “tail end” of the second peak of COVID-19 cases in Victoria. At the peak of the second wave (July 30, 2020), cases in the state peaked at 723, with national totals also peaking on the same day at 745 [15]. This was considered an opportune moment to prospectively source participant lived experience, as there was time to reflect on the height of this second peak while still remaining current in participant memory. All study interviews took place between September 1, 2020, and October 6, 2020. The hospital in which the study was conducted was one of the most impacted by COVID-19 in the state with comparatively high numbers of both patients treated and staff infections. The state reached 0 new infections on October 31, 2020, and this was maintained for 19 days in a row, marking the end of the second wave.

### Procedure

One-on-one telephone and video conference interviews were conducted with eligible participants after written consent was obtained. Semistructured interviews contained 10 items which were a combination of open- and closed-style questions. Questions were adapted from existing literature [12,16,17]. Depending on participant responses, standard prompts or follow-on questions were explored with participants (see Multimedia Appendix 1). Questions were presented in a predefined order, with responses audio recorded to facilitate transcription. Interviews were conducted by CF and EB, both of whom are female, senior clinician psychologists with experience in the conduct of qualitative research. Neither interviewers had worked as direct team members with any respondents, nor had regular clinical involvement with the perioperative department. Interviews were conducted with participants at an agreed upon time and location, with 3/9 participants (33%) opting to conduct the interview on working-from-home days, and the remainder while on-site at the Royal Melbourne Hospital. All participants consented to interviews being audio recorded. This study was approved by the Melbourne Health ethics committee HREC/63609/MH-2020. Participants were informed of their right to withdraw and advised that all data would be collected and disseminated in a confidential manner.

### Statistical Analysis

Transcription was completed by an independent researcher (TW) and analyzed using NVivo software (QSR International). To ensure consistency in transcription, an orthographic notation system outlined by Braun and Clarke [18] was adhered to. As such, transcription encompassed “verbatim” responses in addition to nonverbal expression such as laughter and pauses. Cross-checking of 3/9 (33%) of interviews was undertaken by CF to ensure consistency and quality in transcription. The interlistener consistency for the transcriptions was high (4363/4443 words; 98.19% consistency).

Inductive thematic analysis was undertaken to analyze data due to its flexibility to manage small data sets and established guidelines for use [19]. An epistemological approach of critical realism was adopted when exploring and classifying themes, an approach which assumes that participant language reflects lived reality while acknowledging the influence of society and culture on that reality [18]. Codes were identified from transcribed materials which allowed for the establishment of a coding structure. This coding structure was used to cross-code 3 transcripts to ensure reliability [20]. Themes were then collaboratively developed by the research team to ensure findings represented lived experiences [21]. The consolidated criteria for reporting qualitative research guidelines [22] were used to guide reporting in this study.

## Results

### Participants

A total of 15 staff registered their interest to participate in the study, with 9 agreeing to participate when contacted directly by the interviewers in the research team. The distribution of staff registering interest was 6, 7, 2, and 0 across the 4 monthly registration opportunities. Thus, the decision was made to cease recruitment after month 4, as it was anticipated all staff wanting to participate would have registered interest by this time. This was also mirrored by the diminishing registration rates as the months progressed.

Participants were 9 staff members employed in the perioperative department of the Royal Melbourne Hospital. Nursing staff (n=4) encompassed scrub nurse, scout nurse, clinical nurse specialist, nurse manager, with staff working across roles in some instances. Medical staff (n=5) included anesthetists, neurosurgeons, and orthopedic surgeons.

The sample included a high proportion of senior, experienced staff, with an average of 19.5 years of clinical experience. There was a fairly even split between gender identity (5/9, 56%) female), with no participants identifying as nonbinary, transgender, or having a different gender identity.

### Qualitative Findings

**Figure 1:**
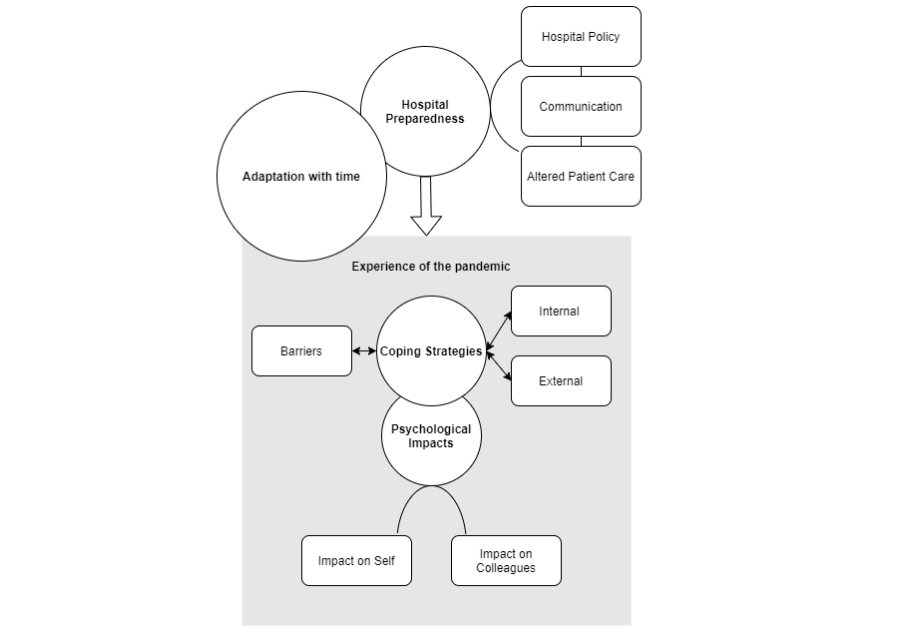
Visual representation of themes and subthemes.

Data saturation was calculated in line with Guest and colleagues [23] with a new information threshold set at ≤5%. Implementing a run length of 2 and base size of 4, data saturation was achieved at interview 6^+3^. Thematic analysis revealed 4 overarching themes: *hospital preparedness to respond, psychological impacts, coping strategies,* and *adaptation with time.* A number of subthemes were also identified and are presented below. The themes and subthemes and their relationships with one another are presented in Figure 1 and are explored with supplementary quotes to assist in interpretation. Quotes have had repetitive words, vocalized filler words (umm, err, ah, etc.), and silent pauses removed for fluency of reading. Parentheses at the end of the quotes provide participant position in the hospital.

As seen in Figure 1, several external factors were conceptualized to impact the experience of the pandemic at a personal and team level. On the former, coping strategies were constructed as a major theme in the experience of the pandemic, with subsequent implications for the psychological impacts of the pandemic. Adaptation with time occurred in parallel to external and personal experiences, with change and growth evident in both contexts.

### Hospital Preparedness

The first emerging theme related to the preparedness of the hospital for the COVID-19 pandemic. This theme related to perception of hospital readiness to face the pandemic and encompassed a number of subthemes illustrated in Table 1. We conceptualized this theme in a top-down fashion, with early changes in hospital policy and communication of these changes having associated implications for patient care. The first subtheme of *hospital policy* was conceptualized by 3 primary factors: readiness to respond, COVID-centric response, rapid policy change, importance of leadership, and response to mental health. There were differing viewpoints regarding the hospital response preparation, with many finding the rapid changes difficult to manage or navigate. Difficulties with unclear lines of leadership, and a perceived lack in the support for mental health well-being provided were also described.

Under the subtheme of *communication*, the method of communication and the information sources were the major factors. The methods of communication were reported to be unclear and inconsistent, while departmental meetings and peer consultation were deemed more useful than electronic online communication methods. *Altered patient care* was the third major subtheme, incorporating the factors of patient implications, changing work roles, and resource availability. Participants were concerned about the impact on care for patients with non-COVID–related health needs. They reported a number of changes to their way of working and projects, concerns about moving areas, and having no work. Comments around resource availability were varied, with several reporting no concerns and others raising issue with the supply and suitability of PPE.

**Table 1:** Theme: Hospital preparedness.

Subtheme and factors			Description		Quotes
**Hospital policy**					
	Readiness to respond	Participants’ perceptions of overall hospital preparedness. While some participants felt confident in the hospital’s capacity to respond, others cited concerns.		*...it personally made me feel confident that the hospital was well prepared because we got on the front foot early and were preparing for the worst and hoping that it wouldn’t happen* [nursing] *I always felt like the hospital was always behind the eight ball when it came to particular things* [medical] *...in the first wave I found that they were a couple of weeks behind a lot of other hospitals* [medical]	
	COVID-centric response	Participants reported concern at the COVID-centric nature of hospital policy, with other areas of clinical practice falling to the wayside.		*...it was very ICU^a^ centric, very ED^b^ centric they made assumptions that theatre would just not be doing anything....they were very focused on one or two areas instead of the whole organization* [nursing] *...it didn’t really take into account the need for us to continue to look after non COVID patients* [medical]	
	Rapid policy change	Rapid changes in policy in the first wave were described by many participants which resulted in workplace stress and confusion.		*in the early stages because of the changes happening so rapidly I think it created a lot of confusion and...there are a lot of people…it was quite stressful* [nursing] *...processes had to escalate quite quickly and changes and decision making at times*[medical]	
	Importance of leadership	In response to rapidly changing procedures and policies, participants highlighted the importance of leadership in managing the dynamic workplace and resultant adverse implications of fractured leadership.		*...sometimes there were potentially too many cooks in the kitchen with some of the decisions and...who was sort of taking the lead and that I guess that ruffles feathers and it leaves a lot of people confused* [nursing] *the leadership across the state and the hospitals have not been clear about what they require from people because most people in health will do whatever you ask as long as they are clear of what you’ve asking and… no one really making a decision because at the end of the day even if the decision is not quite right it’s better to have one than not have one at all* [nursing]	
	Response to mental health	While participants reported changes in hospital policy to manage patient flow and procedures, some commented on weaknesses in the hospital’s response to mental health.		*...other organizations did a lot more to bring wellness on site than the Royal Melbourne Hospital did...for instance our EAP^c^ people weren’t on site where as Western Health insisted that they have people on site where they could meet with people face to face in a socially distanced way we didn’t do that our wellness team would not come to any clinical area so even when one of our staff died they would not come to the area they said we will Zoom Webex people and it’s like ‘what, no thanks they don’t want some person on a screen talking about it its impersonal’* [nursing]	
**Communication**					
	Method of communication	While most participants did feel aware of the hospitals’ response to the pandemic, the methods by which this was communicated was cited as a common source of stress and confusion among teams.		*the only thing that I found in the early days was when we would have other departments come into our area and telling us about changes in PPE^d^ before our leadership had a chance to tell us and I think that was that caused a lot of the early anxiety around things* [nursing] *trying to get that clarity about what was happening yeah it was quite challenging at the time* [nursing]	
	Information sources	Participants primarily highlighted departmental meetings and informal peer consultation as useful means of information transfer. By contrast, participants reported online platforms (specifically Workplace and the hospital intranet) as less useful.		*I was very aware because we were having frequent departmental meetings and updates* [medical] *they used Workplace which I think was a useless tool I think this needed verbal communication not just posting reams of documents on a webpage you know like Facebook people just don’t have the capacity read than and the capacity to even understand what’s changed from day to day* [nursing]	
**Altered patient care**					
	Patient implications	This subtheme revealed concerns that staff had about changing workplace policy and procedure on patient care during the pandemic.		*...everything is slower you know getting surgery is slower if you’ve got an emergency patient it is slower to get them into the operating theatre and you are worried that they are deteriorating while we are all doing the COVID thing* [medical] *everything is slower you know getting surgery is slower if you’ve got an emergency patient it is slower to get them into the operating theatre and you are worried that they are deteriorating while we are all doing the COVID thing* [medical] *other wards...were looking after patients they’d never had to look after before because the normal wards that look after them had gone hot you know there was so many things happening* [medical]	
	Changing work roles	While some participants felt their role had not changed substantially during the pandemic, others did communicate changing work roles, with many citing a reduction in work capacity.		*we dropped the amount of theatres at the beginning and there was kind of just a lot of people floating around and I think that added to the stress because people were like what if I lose my job what if I like I get deployed to aged care I haven’t done I haven’t done age care nursing in like ten years and people were just quite unsure* [nursing] *...definitely a lot things that we did went on the backburner* [nursing] *I sat there and twiddled my thumbs and did nothing* [medical]	
	Resource availability	There was a range of responses in the area of resource availability, with some feeling well-resourced, while others raised concerns about the suitability and availability of PPE		*I’ve never been concerned for my ward or the hospital that we will ever have a shortage of PPE* [nursing] *I never at any point at the Royal Melbourne Hospital felt as though I didn’t have what I needed* [medical] *I’ve never ever felt happy with the N95 masks that they’ve had and I remember at the start of this pandemic when we were starting to wear N95 masks and I said to people then these masks do not fit me I do not feel safe in these masks* [medical] *we ran out of N95 masks quite quickly and as an anaesthetic nurse you were like oh I’m in the head end like I like I’m most likely to be exposed to this what if I get it* [nursing] *having the right correct PPE at times it hasn’t always been ideal not necessarily the correct sizes of masks available not the best ideal gowns available* [nursing]	

^a^ICU: intensive care unit.

^b^ED: emergency department.

^c^EAP: employee assistance program.

^d^PPE: personal protective equipment.

### Psychological Impacts of the Pandemic

This theme related to perceived psychological impacts during the pandemic on self and on colleagues as the 2 major subthemes (Table 2). While many participants did not report needing psychological support themselves, all respondents described seeing psychological impacts in their colleagues. Similarly, many participants reported identifying behavior change in colleagues which was interpreted as being driven by anxiety and stress. The most commonly reported emotion was anger, and other factors identified were the need for psychological support, the spectrum of emotions perceived or experienced, fears of infection, the omnipresent nature of the virus, emotional contagion, and the need for calm.

**Table 2:** Theme: Psychological impact of the pandemic.

Subtheme and factors		Description	Quote
**Impact on self**			
	Need for psychological support	This theme related to self-perceptions of the need for psychological support. Most participants reported not requiring nor actively seeking psychological support.	*...it didn’t get to a point where ahh I thought I needed it* [medical] *...I just I felt like maybe other people needed it more so I just avoided it* [nursing]
	Spectrum of emotion	There were a *range of emotions* experienced by staff which occurred on a spectrum.	*I was just surprised at how upset everyone was by it all* [medical] *I haven’t at any point felt particularly stressed* [nursing]
	Anger	Interestingly, the most commonly expressed emotional response to the pandemic by participants was anger. Of note, the anger originated from causes external to immediate teams.	*I started out being sort of angry and agitated about things and you know* [medical] *I don’t think I was stressed I was angry I still am angry* [medical] *I had a couple of weeks where I was just like really angry really really angry all the time and its hard not to bring that home* [nursing]
	Fear of infection	A number of participants described a fear of catching COVID-19, mostly in relation to bringing the virus home to family and friends.	*...that was quite draining I think everybody early on just felt shattered at the end of every day because they were mindful of the fact they could be infected at any point we didn’t know which patients were likely to be infected or not infected it was a mystery at that level and you know the kind of weight on you personally that you might get infected might get sick might bring it home to your family* [medical] *the main thing that I was worried about was actually bringing the virus home I mean that worried me quite a lot* [medical]
	The omnipresent nature of COVID-19	The all-present nature of the virus was cited as a contributing factor to overall emotional state.	*I used to come home and like literally just strip off at the back door and then run for the shower you really needed that bit of a wind down because COVID was following you all the way home* [medical] *there was a few months back where I had you know extended family members calling me every day to say ‘are you okay we are really worried about you are you okay’ and sometimes the constant questions even though they were coming from a good place I was trying not to think about it when I went home and trying to sort of switch off* [nursing]
	Emotional contagion	Bridging the impact of self and colleagues was the subtheme of emotional contagion. This described the spreading of fear among colleagues which had secondary implications on participants mental health.	*while I was waiting I was just having a conversation with one of the nurses over in theatre and she was kind of talking about the numbers overseas and that in two hundred people working in peri-op one of us was going to die and it was just not something that I needed to hear at 3am and as my mind wasn’t even sort of thinking about that kind of thing I was trying not to* [nursing] *there was nervousness in the air...* [nursing] *they were basing it on a New York situation which we weren’t going to be like New York or Italy and that was very clear from the outset but the panic that ensued through groups of people made It very difficult for people to think rationally and put together a plan for theatre* [nursing]
	Need for calm	In response to emotional contagion, several participants described the need for a calming influence in the workplace, a role which many spontaneously adopted themselves.	*I kind of almost took on a role of being quite calm about it all you know because we can’t all be sort of running around and panicking* [nursing] *I sort of after two weeks had just had enough and told my nursing staff that they were not to participate in the panic with medical staff anymore or the executive and we had to sit down and put a plan into place that was actually going to lead our staff and function through the pandemic* [nursing]
**Impact on colleagues**			
	Behavioral impacts on colleagues	Many participants identified behavioral changes in colleagues as the primary indicator for distress.	*I have felt that some of my colleagues have gone off the rails a little bit in various ways* [medical] *struggling to communicate with various tasks or even just expressing how unsure they were with the masks...you could tell the nervousness like a bit fidgety like a bit sort of racing around sort of thing* [nursing] *this pandemic that has just knocked people out so now you’ve got low energy low morale* [nursing] *reading between the lines in terms of what they are posting online social media what they are texting and a little bit about their behaviour at work...I suspect that some of them have decompensated a little bit* [medical]
	Anxiety	Participants described observing stress and anxiety among colleagues	*you could see people were getting stressed the anxiety was definitely building for staff* [nursing] *there was some people that I felt were quite overwhelmed or looked a bit anxious at times* [nursing] *the anaesthetist probably whose anxiety has been probably not managed well and been extremely high I think has reflected of the lack of recognition of the risk in their workforce group* [nursing]
	Perceived inequality	Perceived inequality between professional groups and wards was cited as a contributing factor to emotion among a team.	*equal recognition is important just because you weren’t in a hot ward it didn’t mean you weren’t working* [nursing] *that’s been really palpable you know through the nurses cause the doctors would say to them ‘ohh why don’t you just work from home just stay at home or just do this were only doing half time here and half time at home’ and its like were not allowed to they don’t see that they have a privilege that others don’t have* [nursing]

### Coping

The theme of *coping* described the various coping strategies used by participants to manage the pandemic (Table 3). These were conceptualized as being either external or internal in nature. More participants reported choosing to use external coping tools (eg, exercise, family, and friends) than internal coping (eg, mindfulness, distraction). Also encompassed under this theme were barriers to using coping strategies that had previously worked, largely stemming from restrictions due to lockdown.

**Table 3:** Theme: Coping.

Subtheme and factors		Description	Quote
**Internal coping**			
	Distraction	Participants described using distraction as a management tool.	*we’ve had some renovation things going on at home as well so that was quite distracting* [medical] *when I went to do some shifts in ICU^a^, I found it was a much more controlled environment and because I was learning new skills and doing something it was a good distraction* [nursing]
	Mindfulness	Use of mindfulness and meditation-based strategies was described by some participants; many using apps to support implementation. Yet other participants reported that these strategies had not been effective.	*I also utilise a lot of online resources a lot of the apps and things that are out there like your smiling mind app and mindfulness and things like downward down dog so that’s the yoga thing but they’ve also got a lot of relaxation things in there as well so I know a lot of apps* [nursing] *...things like meditation just haven’t worked for me because they just don’t on a personal level* [medical] *I had a few weeks there like every night I would come home and I would just sit down and I would put one of those meditation apps on and just lie there for about an hour and just try and calm down and that was quite useful* [medical]
	Mindset	Adjustment to personal mindset was noted to be a helpful internal coping strategy.	*I think the ups and downs of your day I found you have to have the mindset of just take it day by day...otherwise you do get bogged down with thinking about how long it has been...that’s why I am all surprised when people say it has been six or seven months because I can’t I haven’t looked back to when this really all started* [nursing] *like most nurses do I’ve just accepted this is how it is and get on with it* [nursing]
	Role of experience	The role of clinical experience was perceived as a mitigating factor in the experience of stress, with a number of participants highlighting higher levels of distress among more junior staff.	*I think it does come with experience in our environment and knowing where to go to find the information and being a bit more resourceful yourself I think some more junior casual staff probably felt a little bit more at a loss about what’s happening it’s definitely challenging if you aren’t at work for a few days and you come back and your kind of like what’s new what’s happened what’s changed* [nursing] *there were sometimes more junior staff who were getting a bit stressed about all the changes* [nursing]
	Gratitude	A number of participants described feeling grateful for having a job in a time of economic turmoil.	*I’m very grateful that I have a job and I’m grateful that even though I’m coming to the hospital I still get to be able to have face to face with people every day which is a thing that a lot of people don’t have so even though you sort of going in to what you feel is often an unsafe environment the fact that you actually get to still see your colleagues and talk with patients and do all that sort of stuff that’s been good* [medical]
**External strategies**			
	Entertainment	Entertainment was regularly cited by participants as an external coping tool.	*when I was home I played music more you know watched a lot of Netflix and stuff like that* [medical]
	Work as a social outlet	Work was cited as a social outlet. Connection with colleagues played an important role in coping. Similarly, the absence of continued face-to-face contact was cited as a barrier to work engagement.	*I live alone so I found that coming to work was my social life which was actually really good to have* [nursing] *I think a lot of people who you know they probably find coming to work a bit of a relief from being at home all the time* [medical] *being able to go to work and just chatting to your colleagues that’s a that’s a big thing* [medical] *collegiality professional contact is actually quite important and if you just go to work do the work and go home and don’t talk to anybody else its actually rather dull and not very interesting* [medical]
	Limiting media intake	Limiting media intake was reported as a strategy for coping	*I literally turned off because…I’m a bit of a news person I like to keep up with the news and stuff like that but I would literally turn it off I wouldn’t watch any news I wouldn’t read any newspapers cause It was just total twenty four seven saturation about this* [medical]
	Exercise	Exercise was cited by many participants as a helpful external strategy for coping during the pandemic.	*try and exercise that’s usually my best way when I’m stressed* [nursing] *I am a bit of a compulsive exerciser...so you know I’ve got a lot of kind of other things that kind of keep me interested outside of work* [medical] *I’ve managed to get the exercise in that I normally would have done I’m probably healthier physically than I’ve been in a long time* [medical]
	Family and friends	Family and friends played an important role in coping during the pandemic for many participants.	*my coping strategies are I like to stay in touch with people so that’s you know being in touch with family and friends and checking on them* [nursing] *I’ve actually been in contact with a lot more friends and friends from overseas...old friends from school who we haven’t really caught up for ages* [medical]
	Team response to stress	Strong team bonds and a self-initiated team response were implemented to help manage emotional distress during the pandemic.	*we were really good at looking after our little family here on the ward like we all know each other very well and we know when someone’s not right* [nursing] *Yeah I think it’s bought us together* [nursing] *We tried to implement just fun things at work like you know like whoever’s got whatever colour on gets a prize for the day or whatever you know sort of fun things they’ve started a cahoot morning quiz thing and just a few things like that* [nursing] *I felt that almost I was the you know trying to get some of these people aside and talk as though I was being the counsellor in a way you know just giving people the opportunity to talk and stuff like that* [medical]
**Barriers**			
	Lockdown	The imposed restrictions on movement and activity were cited as a barrier for engaging in previously used coping strategies by a number of participants.	*I’m struggling a bit not being able to see my family* [nursing] *you know obviously living alone I couldn’t see my family members for a long time* [nursing] *unfortunately a lot of coping strategies and things I did have got shut down because of the pandemic* [nursing]

^a^ICU: intensive care unit.

### Adaptation With Time

The final theme, *adaptation with time*, reflected change and growth over time, and encompassed all other themes. Within this theme, change was described at both a hospital level and a personal level through psychological adaptation and personal growth (Table 4).

**Table 4:** Theme: Adaptation with time.

Factor	Description	Quote
Hospital adaptation with time	Many participants reported a rapid adaptation of the hospital’s response to the pandemic over time. This was reported in the context of the preparedness for the pandemic at the second wave relative to the first wave.	*I think as time went on we got kind of trouble shooted and it it’s become a lot smoother now* [nursing] *they were weeks ahead dramatic improvement in the response early response to the second wave compared to the first wave* [medical] *pretty rapidly we marshalled a kind of workforce to deal with the various aspects that needed to be done* [medical]
Psychological adaptation with time	Unlike the linear improvement in the hospital’s response, participants described a cycle of emotional change over time.	*so far it’s kind of been a fairly predictable it’s almost like the stages of grief in a way that’s how I feel about it I think that I am kind of at the flat somewhat resigned point now I haven’t reached a point of kind of acceptance I’m sort of in the depression phase a little bit* [medical] *I’ve mostly coped well but there are days where I’ve been emotional and not really understood why and I think it’s probably I’ve just not processed what’s going on* [nursing] *I think I’ve just become a bit more passive in my work and you know not trying to rock the boat too much and although at times I felt that’s been the wrong thing to do its just psychologically for me it’s just a better thing for me to do at the moment not to rock the boat and get angry and upset and just do what you told to do and try and do your best that’s about it* [medical]
Personal growth	A number of participants identified personal growth during the pandemic, with some also reporting changes in life priorities as a result.	*I also feel proud that I am able to deal with this now* [nursing] *I’ve had more contact with people since the pandemic than I did before the pandemic because we were very concentrated on our own lives we used to do a lot of travelling and stuff like that so its actually been a bit of a shift in perspective* [medical]

## Discussion

### Principal Results and Comparison to Prior Work

This study explored the psychological impact of the COVID-19 pandemic among frontline HCWs in the perioperative department of a large, tertiary trauma hospital in Melbourne, Australia. Nine qualitative interviews were conducted with frontline HWCs at the tail end of a second peak of infections in the state of Victoria, Australia. The local environment was a health care service with a high level of exposure to the virus, relative to other services in the state and country. This study is important as it aligns with a recent position paper authored by world-leading experts that reinforced the importance of capturing lived experience when characterizing mental health implications [4]. Qualitative analysis indicated 4 main themes from the data: *hospital preparedness to respond*, *psychological impacts*, *coping strategies*, and *adaptation with time*.

Participants described several emotional states in response to the pandemic, including anger, anxiety, and gratitude, along with psychological concepts including the impact the team and hospital response had on staff emotional well-being and psychological growth. The predominance of anger in our findings is disparate from existing COVID-19 research which has largely found implications for stress, anxiety, and depression [2,5-7]. Yet, findings are consistent with literature from the SARS crisis which highlights anger as a key manifestation of fear and uncertainty [24,25]. Termed as “state anger,” this emotional expression has been purported to be an indicator of underlying emotional distress [24]. It may be that the timing of data collection impacts upon the manifestation of such underlying anger. Notably, participants in our study were interviewed at the tail end of a second and significant wave of infections, with high numbers of infections and associated quarantine requirements among staff. Given this, it may be that anger is an initial emotional reaction experienced by HCWs who are yet to fully process the emotional state underpinning acute experiences. Divergence may also be explained, at least in part, by the professional demographics of HCWs in this study. Unlike Sun and colleagues [12] and Munawar and Choudhry [14], we interviewed a primarily senior and highly experienced sample. It is possible that the nature of these senior work roles and experience of participants lend themselves more intuitively to concern about decision making and resultant implications for staff, rather than the individual experience of distress per se. In support of this notion, several participants in our sample highlighted the unique vulnerability of more junior staff in responding to the pandemic.

Interestingly, despite all participants observing the need for psychological support in colleagues, most indicated that they themselves did not require formal psychological input. Findings are consistent with extant literature, which highlights the perceived stigma around disclosing mental health concerns among health care professionals [26,27]. Reasons cited for nondisclosure have been reported to include anticipated fears of damage to future career prospects and professional standing [28], underpinned by feelings of shame and professional failure [26]. Importantly, Hassan and colleagues [28] found that the reluctance to disclose mental health concerns persisted despite years of clinical experience. Taken together, findings allude to a pervasive stigma associated with mental health among HCWs and reinforce the need for a hospital-wide approach to managing the same.

Hospital preparedness to respond was cited as a contributing factor to perceived emotional stress among participants. Inadequate PPE was noted as contributing to work-related stress, including the fear of personal infection and subsequent likelihood of spreading infection to family and friends. Findings are consistent with existing literature, with shortages of PPE hypothesized to underscore the onset of mental health symptoms [29]. Similarly, trust in infection control procedures and PPE were shown to be negatively correlated with emotional exhaustion and anger among frontline HCWs during the SARS crisis [30]. PPE shortages have been found globally, with the COVID-19 pandemic surfacing systemic issues of supply in response to overwhelming demand [31]. Findings of our research highlight the need for strengthened coordination and dissemination of appropriate and well-fitted equipment to ensure frontline staff are adequately protected.

Underscoring perceived hospital preparedness was inconsistent communication of rapid policy changes, particularly evident in the early stages of the pandemic. The enormous flow of information was unprecedented and was described as originating from a variety of sources, including informally through news media and communication with colleagues. Even when occurring on more formal mediums such as through the hospital Workplace platform, participants described feeling overwhelmed by the amount of information. Importantly, failures to ensure consistency of messaging resulted in emotional contagion and spreading of misinformation through staff in our study, which further exacerbated mistrust, stress, and anxiety. Regulation of communication under such demanding circumstances is undoubtedly challenging. However, our findings highlighted the importance of early, efficient, and consistent communication through regulated means, disseminated from one distinctive leadership group, in better containing the narrative and minimizing the likelihood of miscommunication.

Participants in our study demonstrated remarkable resilience. Despite barriers to implementation due to lockdown, participants continued to use a range of internal psychological coping strategies and external tools in response to growing workplace demands. On external coping strategies, many participants reported using exercise, entertainment, and work as a social outlet to manage stress. Internal psychological strategies of distraction, mindfulness, and positive mindset were also commonly implemented. Teams also demonstrated clear responses to stress, including the spontaneous adoption of “fun” work activities and associated enhancement of team solidarity. Our findings are consistent with Sun and colleagues [12] who reported both active and passive psychological adjustments in response to the pandemic.

Many participants acknowledged that both the hospital response and their own psychological state were not static during the pandemic, but evolved overtime. This was relative to both the stage of pandemic, regarding chronological duration and infection rates, and the different psychological stages of processing and managing the situation. A recent meta-synthesis of frontline worker perceptions of working in a pandemic highlighted personal and professional growth as an emergent theme [32].

### Limitations

This study is not without limitations. Although generalizability is not the goal of qualitative analysis, the sample was taken from a specific team within a single hospital in Melbourne, Victoria, Australia. As such, it may not be transferrable to HCWs from other clinical areas, disciplines, hospitals, or states. The sample size was also small and may not fully reflect the views of all of the perioperative teams. This may be the case particularly for junior and less experienced HCWs in the team who were underrepresented in the self-selected participants. However, this is one of the first studies to qualitatively examine the psychological impact of COVID-19 on HCWs in Australia. Further insights into the psychological response of this team will also be disseminated on completion of a longitudinal survey currently being undertaken.

Several recommendations to assist HCWs’ psychological functioning for organizations managing pandemic situations can be offered from these data. As noted, clear and consistent communication regarding changes to working practices and procedures is likely to facilitate staff feeling informed and assured in the response of the service. Centralized and visual leadership within the organization, particularly when communicating the service response, is also recommended. Visible and available organizationally promoted staff well-being measures are desired by staff, as well as accommodations to working procedures that are as equitable as possible, across professional disciplines. Acknowledgement of the wide variety of psychological responses that people have to stressful pandemic situations may also be advantageous, and a normalization of these responses, so that individuals do not feel unnecessary concerned about their own response, or pressure to respond in a particular way, may be needed.

### Conclusions

The psychological impact of stressful and risky situations for HCWs, particularly during periods of prolonged stress, should not be overlooked. While frontline staff and hospitals have rapidly marshalled a response to managing the virus, relatively less consideration was seen regarding staff mental health in our study. Many participants described an emotional response to the pandemic, though barriers in help seeking remain evident. Hospital preparedness was cited as a contributing factor to emotional response, including availability of well-fitted PPE and concerns with communication of rapidly evolving policies. Encouragingly, participants reported using a varied number of coping mechanisms, with adaptation over time evident in response to local barriers. Findings highlight the vulnerability of HCWs in response to the pandemic and reinforce the need for a coordinated approach to managing mental health.

## Appendix

Multimedia Appendix 1 Question schedule.

## Abbreviations

HCW: health care worker
PPE: personal protective equipment

## References

[1] Li Q, Guan X, Wu P, Wang X, Zhou L, Tong Y, Ren R, Leung KS, Lau EH, Wong JY, Xing X, Xiang N, Wu Y, Li C, Chen Q, Li D, Liu T, Zhao J, Liu M, Tu W, Chen C, Jin L, Yang R, Wang Q, Zhou S, Wang R, Liu H, Luo Y, Liu Y, Shao G, Li H, Tao Z, Yang Y, Deng Z, Liu B, Ma Z, Zhang Y, Shi G, Lam TT, Wu JT, Gao GF, Cowling BJ, Yang B, Leung GM, Feng Z (2020). Early Transmission Dynamics in Wuhan, China, of Novel Coronavirus–Infected Pneumonia. N Engl J Med.

[2] World Health Organization WHO Coronavirus Disease (COVID-19) Dashboard. Secondary WHO Cornoavirus Disease (COVID-19) Dashboard 2020.

[3] Lai J, Ma S, Wang Y, Cai Z, Hu J, Wei N, Wu J, Du H, Chen T, Li R, Tan H, Kang L, Yao L, Huang M, Wang H, Wang G, Liu Z, Hu S (2020). Factors Associated With Mental Health Outcomes Among Health Care Workers Exposed to Coronavirus Disease 2019. JAMA Netw Open.

[4] Morgan C, Rose N (2020). Multidisciplinary research priorities for the COVID-19 pandemic. The Lancet Psychiatry.

[5] Cabarkapa Sonja, Nadjidai Sarah E, Murgier Jerome, Ng Chee H (2020). The psychological impact of COVID-19 and other viral epidemics on frontline healthcare workers and ways to address it: A rapid systematic review. Brain Behav Immun Health.

[6] Department of Health and Human Services (2020). Secondary Victorian healthcare worker (clinical and non-clinical) coronavirus (COVID-19) data 2020.

[7] Pappa S, Ntella V, Giannakas T, Giannakoulis VG, Papoutsi E, Katsaounou P (2021). Corrigendum to "Prevalence of depression, anxiety, and insomnia among healthcare workers during the COVID-19 pandemic: A systematic review and meta-analysis" [Brain Behav. Immun. 88 (2020) 901-907]. Brain Behav Immun.

[8] Shaukat N, Ali DM, Razzak J (2020). Physical and mental health impacts of COVID-19 on healthcare workers: a scoping review. Int J Emerg Med.

[9] Kang L, Ma S, Chen M, Yang Jun, Wang Ying, Li Ruiting, Yao Lihua, Bai Hanping, Cai Zhongxiang, Xiang Yang Bing, Hu Shaohua, Zhang Kerang, Wang Gaohua, Ma Ci, Liu Zhongchun (2020). Impact on mental health and perceptions of psychological care among medical and nursing staff in Wuhan during the 2019 novel coronavirus disease outbreak: A cross-sectional study. Brain Behav Immun.

[10] Huang Y, Zhao N (2020). Generalized anxiety disorder, depressive symptoms and sleep quality during COVID-19 outbreak in China: a web-based cross-sectional survey. Psychiatry Res.

[11] Spoorthy MS, Pratapa SK, Mahant S (2020). Mental health problems faced by healthcare workers due to the COVID-19 pandemic-A review. Asian J Psychiatr.

[12] Sun N, Wei L, Shi S, Jiao D, Song R, Ma L, Wang H, Wang C, Wang Z, You Y, Liu S, Wang H (2020). A qualitative study on the psychological experience of caregivers of COVID-19 patients. Am J Infect Control.

[13] Tan Rong, Yu Ting, Luo Kaiyan, Teng Fen, Liu Yilan, Luo Jian, Hu Deying (2020). Experiences of clinical first-line nurses treating patients with COVID-19: A qualitative study. J Nurs Manag.

[14] Munawar K, Choudhry FR (2021). Exploring stress coping strategies of frontline emergency health workers dealing Covid-19 in Pakistan: A qualitative inquiry. Am J Infect Control.

[15] Department of Health and Human Services Secondary Victorian Coronavirus (COVID-19) Data 2020.

[16] Maunder R, Lancee W, Balderson K, Bennett J, Borgundvaag B, Evans S, Fernandes C, Goldbloom D, Gupta M, Hunter J, McGillis Hall L, Nagle L, Pain C, Peczeniuk S, Raymond G, Read N, Rourke S, Steinberg R, Stewart T, VanDeVelde-Coke S, Veldhorst G, Wasylenki D (2006). Long-term psychological and occupational effects of providing hospital healthcare during SARS outbreak. Emerg Infect Dis.

[17] Corley A, Hammond NE, Fraser JF (2010). The experiences of health care workers employed in an Australian intensive care unit during the H1N1 Influenza pandemic of 2009: a phenomenological study. Int J Nurs Stud.

[18] Braun V, Clarke V (2013). Successful Qualitative Research: A Practical Guide for Beginners.

[19] Braun V, Clarke V (2006). Using thematic analysis in psychology. Qualitative Research in Psychology.

[20] Macqueen K, McLellan-Lemal E, Bartholow K, Milstein B (2008). Team-based codebook development: structure, process, and agreement. Handbook for Team-Based Qualitative Research.

[21] Nowell LS, Norris JM, White DE, Moules NJ (2017). Thematic Analysis. International Journal of Qualitative Methods.

[22] Tong A, Sainsbury P, Craig J (2007). Consolidated criteria for reporting qualitative research (COREQ): a 32-item checklist for interviews and focus groups. Int J Qual Health Care.

[23] Guest G, Namey E, Chen M (2020). A simple method to assess and report thematic saturation in qualitative research. PLoS One.

[24] Fiksenbaum L, Marjanovic Z, Greenglass ER, Coffey S (2006). Emotional Exhaustion and State Anger in Nurses Who Worked During the Sars Outbreak: The Role of Perceived Threat and Organizational Support. Canadian Journal of Community Mental Health.

[25] Mok E, Chung BP, Chung JW, Wong TK (2005). An exploratory study of nurses suffering from severe acute respiratory syndrome (SARS). Int J Nurs Pract.

[26] Galbraith N, Boyda D, McFeeters D, Hassan T (2021). The mental health of doctors during the COVID-19 pandemic. BJPsych Bull.

[27] Knaak S, Mantler E, Szeto A (2017). Mental illness-related stigma in healthcare: Barriers to access and care and evidence-based solutions. Healthc Manage Forum.

[28] Hassan TM, Tran T, Mazhar MN, Doan N, Munshi T, Bajaj N, Groll D, Galbraith N (2016). Attitudes of Canadian psychiatry residents if mentally ill: awareness, barriers to disclosure, and help-seeking preferences. Can. Med. Ed. J.

[29] Lam SC, Arora T, Grey I, Suen LKP, Huang EY, Li D, Lam KBH (2020). Perceived Risk and Protection From Infection and Depressive Symptoms Among Healthcare Workers in Mainland China and Hong Kong During COVID-19. Front Psychiatry.

[30] Marjanovic Z, Greenglass ER, Coffey S (2007). The relevance of psychosocial variables and working conditions in predicting nurses' coping strategies during the SARS crisis: an online questionnaire survey. Int J Nurs Stud.

[31] Burki T (2020). Global shortage of personal protective equipment. The Lancet Infectious Diseases.

[32] Billings J, Ching B, Gkofa V, Greene T, Bloomfield M (2013). Healthcare workers' experiences of working on the frontline and views about support during COVID-19 and comparable pandemics: a rapid review and meta-synthesis. medRxiv.

