# Supplementary material for: The Psychological Experience of Frontline Perioperative Health Care Staff in Responding to COVID-19: Qualitative Study
Source: JMIR Perioper Med. 2021 Sep 29;4(2):e27166. doi: 10.2196/27166 (PMC8483271; doi:10.2196/27166)
Supplement: Multimedia Appendix 1 [file periop_v4i2e27166_app1.docx]

**“It’s like the stages of grief”: A Qualitative Study of the Psychological Experience of Frontline Perioperative Healthcare Staff in Responding to COVID-19**

**Appendix A**

*Interview Schedule*

| Question | Prompt |
| --- | --- |
| 1. Can I start by checking your role and the length of time you have been working here? |  |
| 2. How aware were you of the hospital preparations for the peak of the COVID-19 pandemic? | Where did you access your information about preparation; Was it useful for you; Did the preparations take account of the needs of perioperative staff and the areas in which you work? |
| 3. What did you find to be the main challenges that you (have) faced during the COVID-19 pandemic? | Were they of a professional nature; Did they relate to personal issues; or a combination of these? Can you tell me about them; How you managed these challenges? |
| 4. Did you find that your professional role changed during the COVID-19 pandemic? | If yes, can you tell me what changed during this time; Did it change how you saw your professional role? |
| 5. Do you have any coping strategies to help reduce stress during the COVID-19 pandemic? If so, what are they? |  |
| 6. Did you access psychological support during the COVID pandemic? | If yes, what form did this take? Was it helpful? |
| 7. Were there times during the Covid19 pandemic that you felt like you needed psychological support but didn’t access it? |  |
| 8. Did you notice that colleagues needed psychological support during this time? | If yes, can you give me an example; What action did you or others take?  If no, what supports do you think they may have been drawing on to reduce stress? |
| 9. Did you feel that you had the resources that you needed to do your job during this time? | Equipment and staffing prompt |
| 10. Is there anything else you would like to add that might be helpful as we think through how to assist staff to manage should a similar situation arise again? |  |
